# Elucidation of Molecular Mechanism of a Selective PPARα Modulator, Pemafibrate, through Combinational Approaches of X-ray Crystallography, Thermodynamic Analysis, and First-Principle Calculations

**DOI:** 10.3390/ijms21010361

**Published:** 2020-01-06

**Authors:** Mayu Kawasaki, Akira Kambe, Yuta Yamamoto, Sundaram Arulmozhiraja, Sohei Ito, Yoshimi Nakagawa, Hiroaki Tokiwa, Shogo Nakano, Hitoshi Shimano

**Affiliations:** 1 Graduate Division of Nutritional and Environmental Sciences, University of Shizuoka, 52-1 Yada, Suruga-ku, Shizuoka 422-8526, Japan; s18217@u-shizuoka-ken.ac.jp (M.K.); f16007@u-shizuoka-ken.ac.jp (A.K.); itosohei@u-shizuoka-ken.ac.jp (S.I.); 2 Department of Chemistry, Rikkyo University, 3-34-1 Nishi-Ikebukuro, Toshima-ku, Tokyo 171-8501, Japan; yuta.abinitio.yamamoto@gmail.com (Y.Y.); raja@rikkyo.ac.jp (S.A.); tokiwa@rikkyo.ac.jp (H.T.); 3 Japan Agency for Medical Research and Development-Core Research for Evolutional Science and Technology (AMED-CREST), Chiyoda-ku, Tokyo 100-1004, Japan; 4 Department of Internal Medicine (Endocrinology and Metabolism), Faculty of Medicine, University of Tsukuba, 1-1-1 Tennodai, Tsukuba, Ibaraki 305-8575, Japan; yosshy@md.tsukuba.ac.jp; 5 International Institute for Integrative Sleep Medicine (WPI-IIIS), University of Tsukuba, 1-1-1 Tennodai, Tsukuba, Ibaraki 305-8575, Japan; 6 Research Center for Smart Molecules, Rikkyo University, 3-34-1 Nishi-Ikebukuro, Toshima-ku, Tokyo 171-8501, Japan; 7 Life Science Center for Survival Dynamics, Tsukuba Advanced Research Alliance (TARA), University of Tsukuba, 1-1-1 Tennodai, Tsukuba, Ibaraki 305-8577, Japan

**Keywords:** pemafibrate, SPPARMα, X-ray crystal structure, isothermal titration calorimetry, fragment molecular orbital theory

## Abstract

The selective PPARα modulator (SPPARMα) is expected to medicate dyslipidemia with minimizing adverse effects. Recently, pemafibrate was screened from the ligand library as an SPPARMα bearing strong potency. Several clinical pieces of evidence have proved the usefulness of pemafibrate as a medication; however, how pemafibrate works as a SPPARMα at the molecular level is not fully known. In this study, we investigate the molecular mechanism behind its novel SPPARMα character through a combination of approaches of X-ray crystallography, isothermal titration calorimetry (ITC), and fragment molecular orbital (FMO) analysis. ITC measurements have indicated that pemafibrate binds more strongly to PPARα than to PPARγ. The crystal structure of PPARα-ligand binding domain (LBD)/pemafibrate/steroid receptor coactivator-1 peptide (SRC1) determined at 3.2 Å resolution indicates that pemafibrate binds to the ligand binding pocket (LBP) of PPARα in a Y-shaped form. The structure also reveals that the conformation of the phenoxyalkyl group in pemafibrate is flexible in the absence of SRC1 coactivator peptide bound to PPARα; this gives a freedom for the phenoxyalkyl group to adopt structural changes induced by the binding of coactivators. FMO calculations have indicated that the accumulation of hydrophobic interactions provided by the residues at the LBP improve the interaction between pemafibrate and PPARα compared with the interaction between fenofibrate and PPARα.

## 1. Introduction

Peroxisome proliferator-activated receptors (PPARs) are members of families of nuclear receptors, which play an important role in lipid metabolism. Three PPAR subtypes (PPARα, PPARγ, PPARδ) are expressed in different tissues. PPARα is mainly expressed in metabolically active tissues, such as liver, kidney, heart, and muscle [1]. PPARγ is confirmed in white and brown adipocytes, and PPARδ is expressed in many tissues ubiquitously with lower expression levels compared with the other two subtypes [1]. In this study, PPARα and PPARγ were used as target molecules. For physiological functions, PPARα regulates gene expression involved in peroxisomal and mitochondrial β-oxidation [2], whereas PPARγ modulates the expression involved in energy storage and utilization [1].

Because of these backgrounds, the activation of PPARs would be appropriate to medicate various diseases, such as dyslipidemia and type 2 diabetes. Several PPARα and PPARγ agonists have been developed for now [1]. X-ray structural analysis would be helpful to figure out how the agonists activate PPARs at the molecular level. Many research groups succeeded in determining the cocrystal structures of PPARs with their agonists [3,4,5,6,7,8,9], suggesting that there is a common mechanism for the activation; AF2 interface is stabilized by binding of agonists to ligand-binding pocket (LBP) of PPARs ligand binding domain (LBD) [8], and this stabilization makes easy for PPARs to recruit coactivators. Although the activation mechanism appeared to be common among PPARs, LBP configuration is different among PPAR subtypes [10]. Thus, the binding affinity of PPAR agonists toward each PPAR subtypes can be regulated by changing their chemical structure [8]. Among the agonists, selective PPAR modulators that can activate PPARα (SPPARMα) [11] and PPARγ (SPPARMγ) [12,13,14,15] selectively are expected to be alternatives of conventional PPAR agonists because they can reduce side effects. Currently, thiazolidinone [16,17,18] and fibrate [19,20,21,22] compounds have been developed to activate PPARγ and PPARα, respectively; in this study, we focused on fibrates.

In the fibrate class, fenofibric acid (fenofibrate) was developed as a PPARα agonist for the treatment of dyslipidemia [23]. In clinical trials, the administration of fibrates could reduce the concentration of triglycerides (TG) and increase the concentration of high-density lipoprotein (HDL) in plasma [24,25]. However, fibrates such as fenofibrate and bezafibrate could not come up with a satisfactory outcome in preventing cardiovascular events in the limited group of dyslipidemic patients presumably due to weak potency and some adverse effects, such as myopathy and renal dysfunction [26]. The development of SPPARMα bearing high potency has been expected to overcome these disadvantages.

Recently, pemafibrate was identified as a SPPARMα and is shown to have strong potency. Pemafibrate was designed through the transactivation analysis of many substituents to improve its potency and selectivity toward PPARα [27]. The activity was confirmed by in vitro and in vivo assay analysis—in cell-based assays, pemafibrate is shown to have over 2500-fold higher potency than that of the fenofibric acid [28]. Gene expression analysis also supported that treatment of pemafibrate activates PPARα [29]. Preclinical study showed that pemafibrate markedly decreases the TG concentration and increases HDL-C levels compared with fenofibric acid [30]. In addition, pemafibrate exhibits no serious adverse effects, such as renal and hepatic disorders [28,31]. Combination therapy of pemafibrate and statin reduces TG levels significantly compared with statin monotherapy by reducing adverse effects [32]. These characteristic activities appear to be brought by unique Y-shaped structures of pemafibrate which consists of carboxylic, phenoxyalkyl, and 2-aminobenzoxasole groups [27]. By using in silico approaches, we suggested earlier that the Y-shaped pemafibrate interacts with PPARα by fully occupying its LBP [33]. However, further studies are necessary to confirm the molecular mechanism of pemafibrate interaction with PPARα due to the lack of cocrystal structure of PPARα with pemafibrate.

In this study, we elucidate the molecular mechanism behind SPPARMα nature of pemafibrate by a combination of approaches of isothermal titration calorimetry (ITC), X-ray crystallography, and fragment molecular orbital (FMO) method. ITC measurements indicated that pemafibrate is selective against PPARα. The crystal structure of the PPARα-LBD/pemafibrate/SRC1 complex revealed a unique binding mode of pemafibrate. Interaction energy analysis by FMO calculation between PPARα and pemafibrate showed that pemafibrate formed many hydrophobic interactions with the residues sitting in the LBP site. Taken together, we confirmed the unique functionalities of pemafibrate based on structural and functional analysis approaches.

## 2. Results

### 2.1. Estimation of Binding Affinity between PPARs-LBD and Two Fibrates

At first, we attempted to indicate that pemafibrate selectively binds to PPARα by in vitro assay. Here, ITC measurement is suitable to estimate the binding affinities. With this approach, dissociation constant (*K*_d_) can be directly determined by monitoring heat generated by binding ligands to the target protein. The ITC measurement also provides the difference in enthalpy (Δ*H*) and entropy (−*T*Δ*S*) values due to complexation, which is helpful in understanding the nature of interactions.

**Figure 1:**
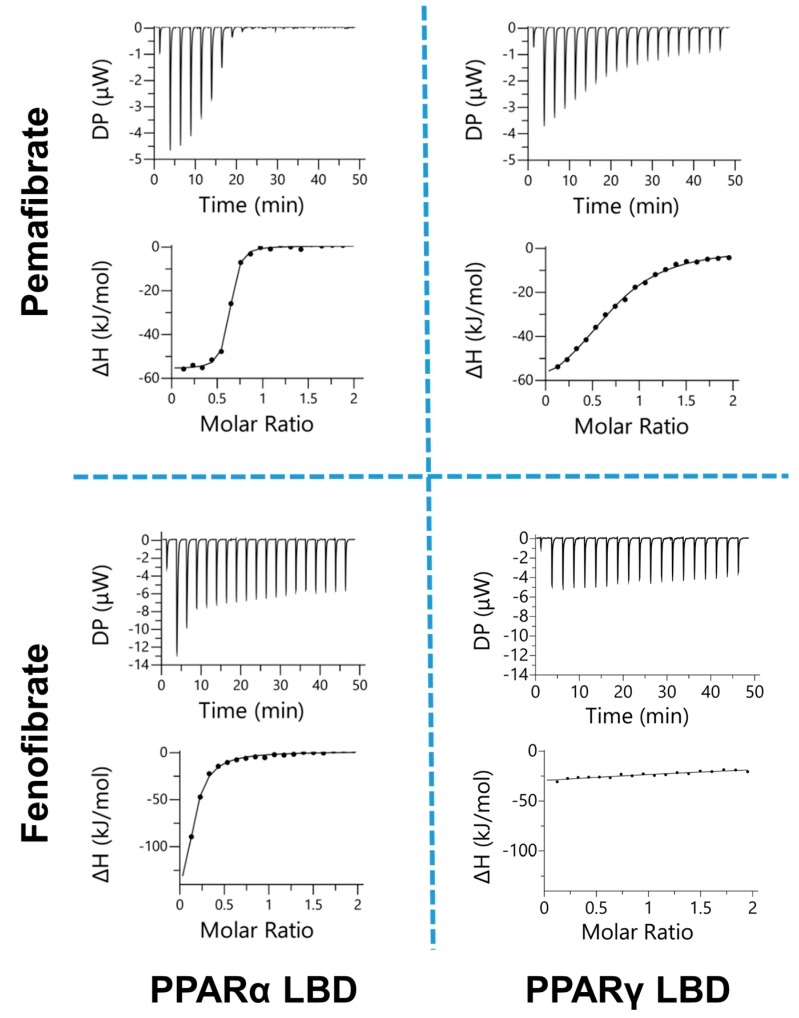
Isothermal titration calorimetry (ITC) analysis of fenofibric acid and pemafibrate binding to peroxisome proliferator activated receptor α (PPARα) and PPARγ ligand binding domain (LBD). The upper and lower panels of ITC data represent the heat signal generated by the binding of each ligand to PPARs in the cells and integration of the signal per injection, respectively. All the relevant parameters are given in Table 1.

In this study, we measured the binding affinities of two fibrates (pemafibrate and fenofibric acid) to PPARs-LBD (PPARα- and PPARγ-LBD) using ITC (Figure 1); as one of the fundamental and important members of the fibrates family, fenofibrate was used to compare the activity difference with pemafibrate broadly [11,30,32,34]. Here, PPARγ-LBD was selected for a comparison of PPARα-LBD because fenofibrate activates PPARα and PPARγ, but not PPARδ [11]. The thermodynamic parameters are shown in Table 1. The binding isotherms depicted in Figure 1 (sigmoidal curve1), suggest that the fibrates bind to the PPARs-LBD except fenofibric acid to PPARγ-LBD. The interaction between fenofibric acid and PPARγ-LBD was too weak to determine the thermodynamic parameters (Table 1). The obtained parameters indicate that pemafibrate selectively binds to PPARα-LBD as expected. In fact, the *K*_d_ value obtained for the pemafibrate/PPARα-LBD complex is 0.13 μM which is around 50- and 70-fold lower than the values obtained for fenofibric acid/PPARα-LBD (7.37 μM, Table 1), and pemafibrate/PPARγ-LBD (9.58 μM, Table 1), respectively.

Here, one question may emerge on how pemafibrate can bind to PPARα selectively. To answer this question, we compared two energetic terms, Δ*H* and −*T*Δ*S*. ΔH represents highly specific interactions, such as hydrogen bond and Van der Waals interactions [35]. −*T*Δ*S* indicates low specific interactions, including hydrophobic interaction [36]. The binding affinity of drug molecules could be improved by optimizing both Δ*H* and −*T*Δ*S* values; in fact, strong statins, such as rosuvastatin, have more favorable Δ*H* and −*T*Δ*S* values than classic statins [35]. The Δ*G* value of PPARα-LBD and pemafibrate, which is a summation of the Δ*H* and −*T*Δ*S* values, was lower than the Δ*G* value of PPARα-LBD and fenofibrate, and PPARγ-LBD and pemafibrate (Table 1). This favorable Δ*G* value of PPARα-LBD and pemafibrate to form strong interactions appears to be brought by the optimization of both Δ*H* and −*T*Δ*S* values. In fact, the Δ*H* value of pemafibrate to PPARα-LBD bears a negative value (−12.3 kcal/mol) as well as other interactions, such as the interaction between PPARα-LBD and fenofibrate, and PPARγ-LBD and pemafibrate. On the other hand, the −*T*Δ*S* value of pemafibrate to PPARα-LBD (3.13 kcal/mol) was clearly improved compared to other interactions (Table 1). The improvement of the interaction between pemafibrate and PPARα-LBD may be due to its two moieties: phenoxyalkyl and 2-aminobenzoxasole groups. This will be analyzed in the following section in detail. These results suggest that hydrophobic interactions, which are characteristic in the pemafibrate/PPARα-LBD binding, improve the affinity of pemafibrate to PPARα.

### 2.2. Crystal Structure Analysis for Pemafibrate and SRC1 Peptide Binding form of PPARα-LBD

**Figure 2:**
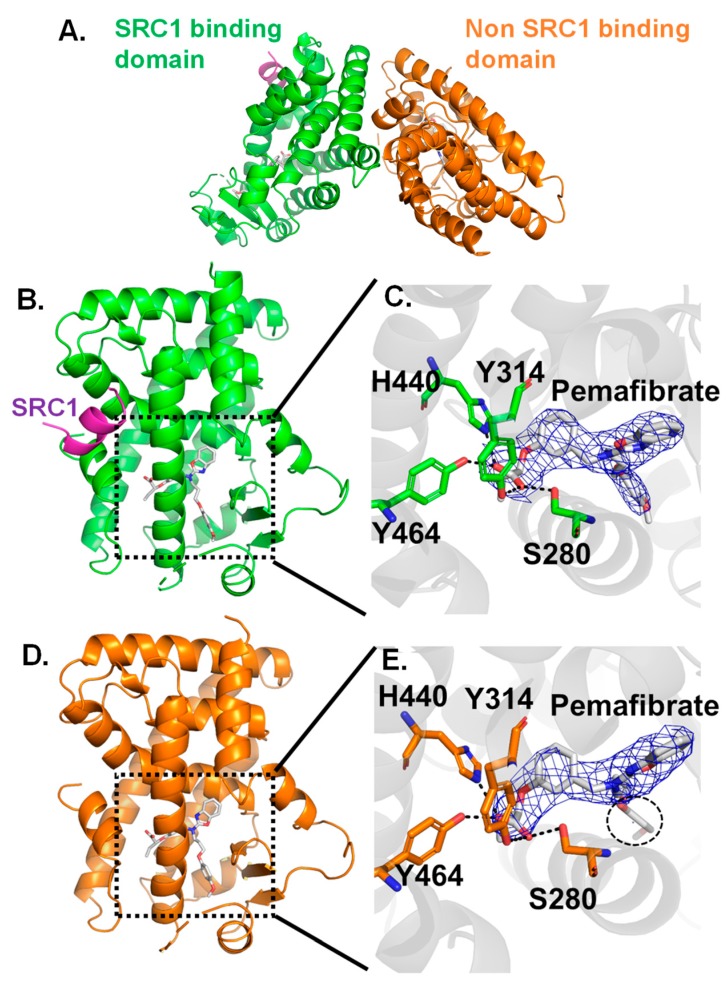
(**A**) Overall structure of PPARα-LBD/pemafibrate/SRC1 peptide. The domain which binds both pemafibrate and SRC1 peptide colored in green, and the domain which binds only pemafibrate colored in orange. Crystal structure of SRC1 binding domain (**B**) and LBP site (**C**). Crystal structure of the non-SRC1 binding domain (**D**) and LBP site (**E**). In both structures, three residues (Y314, H440, and Y464) form hydrogen bonds with the carboxyl group of pemafibrate. The 2*F*_o_-*F*_c_ electron density map contoured at 1.0*σ*.

ITC measurements have suggested that the characteristic hydrophobic interactions would improve affinity of pemafibrate to PPARα. To prove this point from structural analysis, crystal structure of PPARα-LBD/pemafibrate/SRC1 complex is determined at 3.2 Å resolution (Table 2). The structure forms dimer in asymmetric unit, in one subunit the protein binds both with pemafibrate and the SRC1 peptide (green, Figure 2A) while in the other subunit the protein binds only with pemafibrate (orange, Figure 2A). These domains are referred as SRC1 binding domain and non-SRC1 binding domain, respectively (Figure 2).

The structure comparison between SRC1 binding (Figure 2B) and non-SRC1 binding domains (Figure 2D) indicates that overall the structures are almost identical to each other; in fact, the root mean square deviation value for Cα atoms is 0.315 Å, which indicates that no drastic conformational change is induced in the PPARα-LBD structure by the binding of SRC1 peptide. In both of the structures, pemafibrate binds to the LBP of PPARα-LBD (Figure 2C,E). The carboxyl group of pemafibrate forms hydrogen bonding interactions with Y314, H440, and Y464, Figure 2C,E, and these interactions were also observed in other agonists bound to PPARα [3,37,38].

Here, the flexibility of pemafibrate appeared to be changed with or without binding of SRC1 peptide from structure comparison between the domains. In fact, the electron density of pemafibrate is clearly confirmed in the SRC1 binding domain (blue mesh in Figure 2C); the electron density has a Y-shaped form to fill a cavity at the LBP of PPARα (Figure 2C). On the other hand, there is no electron density for phenoxyalkyl group of pemafibrate in the non-SRC1 binding domain (blue mesh in Figure 2E). The high flexibility of the phenoxyalkyl group of pemafibrate would work for favorable interactions with PPARα by changing its conformation depending on the structural changes of PPARα induced by the binding of various coactivators.

### 2.3. Interaction Energy Analysis between Pemafibrate and PPARα-LBD Based on FMO Method

Utilizing the crystal structure of PPARα-LBD/pemafibrate/SRC1 complex, residue-level interaction energies between pemafibrate and residues at LBP of PPARα can be quantitatively obtained through computational analysis. In this study, the quantum-mechanical (electron correlation incorporated) FMO method has been used to calculate the interaction energies. The residue-level interaction energies are estimated through interfragment interaction energy (IFIE) calculations. The calculated IFIEs consist of three energy terms namely, HF-IFIEs, dMP2-IFIEs, and MP2-IFIEs. HF-IFIEs and MP2-IFIEs are obtained by applying the Hartree–Fock and Møller–Plesset perturbation theory, respectively. HF-IFIEs mostly represent electrostatic interactions. MP2-IFIEs are the sum of HF-IFIEs and electron correlation energies, which roughly represent hydrophobic interactions. The dMP2-IFIEs are obtained by subtracting HF-IFIEs from MP2-IFIEs; thus, the term dMP2-IFIEs contains only the electron correlation energies.

**Figure 3:**
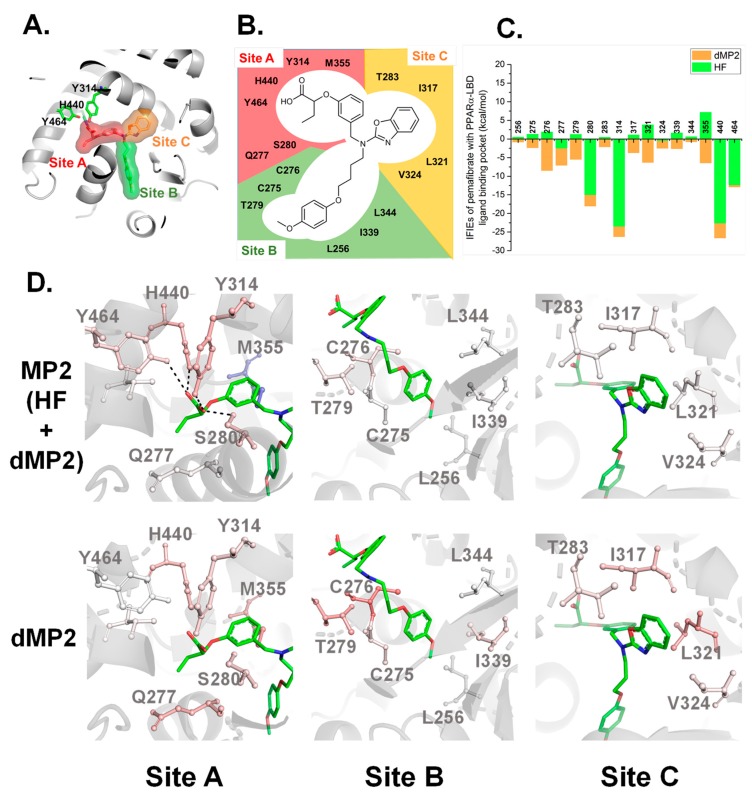
(**A**) Division of the LBP site of PPARα into three sites. The LBP is divided into three sites: site A (red surface), site B (green surface), and site C (orange surface). (**B**) Potential interacting residues at the LBP of PPARα with pemafibrate. A total of 16 residues were located within 3.2 Å distance from pemafibrate. (**C**) Significant interfragment interaction energies (IFIEs) between pemafibrate and LBP residues of PPARα. The HF-IFIEs and dMP2-IFIEs represented in green and orange, respectively. (**D**) Representation of MP2-IFIEs (upper row) and dMP2-IFIEs (bottom row) at the three sites on the crystal structure of the PPARα-LBD/pemafibrate/SRC1 peptide. The structures are colored depending on the magnitude of the IFIEs value. Positive (repulsive) and negative (attractive) IFIEs are colored by blue and red, respectively.

For the convenience to analyze IFIEs, the LBP of PPARα-LBD is divided into three sites depending on the binding of pemafibrate: site A (red surface in Figure 3A), site B (green surface in Figure 3A), and site C (orange surface in Figure 3A), respectively. The three sites are formed by the residues indicated in Figure 3B. Among the six residues forming site A, five residues bear polar groups on their side chains (Figure 3B), suggesting that electrostatic interactions would be formed at site A with pemafibrate. On the other hand, site B and C are mainly formed by hydrophobic residues (eight out of ten residues), inferring that, at these sites, hydrophobic interactions would be the main contributor to form stable interactions with pemafibrate (Figure 3B).

We calculated IFIEs that represent interaction energies between pemafibrate and residues at LBP of PPARα-LBD to show which residues interact with pemafibrate strongly (Figure 3C). In addition, depending on the magnitude of MP2-IFIEs and dMP2-IFIEs, the residues were colored on crystal structure of PPARα-LBD/pemafibrate/SRC1. The figures would be helpful in figuring out the residues that interact with pemafibrate visually. As expected, four residues at site A (S280, Y314, Y440, and Y464) formed strong electrostatic interactions with pemafibrate compared with other residues (Figure 3D, MP2 at the site A). In fact, about 65% of the total MP2-IFIEs (−113.20 kcal/mol) are derived from HF-IFIEs of these four residues (−73.55 kcal/mol) (Figure 3C). The remaining 35% of stabilization would be brought by the hydrophobic interaction (dMP2-IFIEs) between pemafibrate and PPARα-LBD. Evidently, almost all of the residues at the three sites are colored in red when the contribution of dMP2-IFIEs was reflected in the structure (lower line in Figure 3D). This suggested that the accumulation of weak hydrophobic interactions between pemafibrate and hydrophobic residues at the LBP of PPARα also contribute to improving the binding affinity.

## 3. Discussion

**Figure 4:**
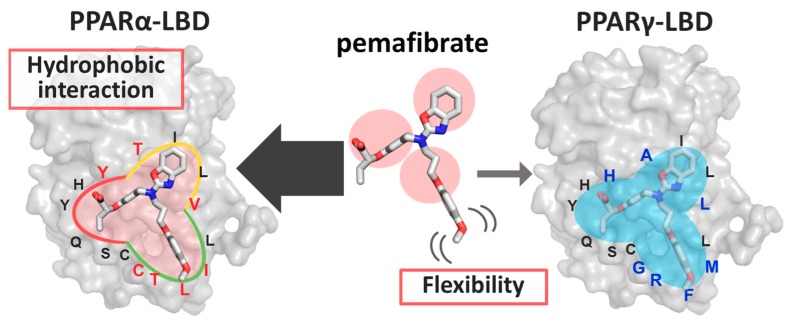
Schematic view of the molecular mechanism behind pemafibrate as a selective PPARα modulator (SPPARMα).

Compared with fenofibrate, the administration of a lower concentration of pemafibrate strongly and selectively activates PPARα. By using the results derived through the combination of analysis of the crystal structure, ITC measurement, and FMO calculations in this study, we propose two plausible reasons for the novel SPPARMα activity of pemafibrate in detail (Figure 4).

The first reason is that the accumulation of hydrophobic interactions formed between several hydrophobic residues with pemafibrate could improve the overall binding affinity between pemafibrate and PPARα. Crystal structure analysis indicated that chemical groups of pemafibrate occupy three sites (site A, site B, and site C) at LBP of PPARα (Figure 2C), whereas fenofibrate can bind to PPARα by occupying two sites from their chemical structure. The difference in the number of interactions would be a reason that pemafibrate works as a strong SPPARMα compared with fenofibrate. Furthermore, this point is well supported by the FMO analysis; around 16 residues at the LBP of PPARα (Figure 3B) form weak hydrophobic interactions with pemafibrate (see dMP2 values in Figure 3C). The results indicate that one-point mutation at the LBP would have only little effect on the total binding affinity of pemafibrate because other residues would compensate for the loss of the interaction by the mutation. It should be mentioned here that our earlier study indicated that, for the case of pemafibrate, five PPARα mutants (C275A, S280A, L321A, I339A, and L344A) remained active (range of 25 to 70%) compared with wild type PPARα [33]. On the other hand, for the case of fenofibric acid, the activity was drastically decreased by the mutations, especially for the I339A and L344A mutants, the activity was hardly detected [33]. Meanwhile, comparing LBP structures of PPARα and PPARγ indicates their sequence differences: C275, T279, T283, Y314, V324, and I339 in PPARα are mutated to Gly, Arg, Ala, His, Leu, and Met in PPARγ, respectively (colored residues in Figure 4). These differences would explain the reduction in the binding affinity of pemafibrate to PPARγ. Of course, we recognize that the activity change was measured only for Ala mutants of PPARα [33]. Thus, we cannot evaluate another possibility on how pemafibrate works as an SPPARMα—less favorable interactions, such as steric clash of pemafibrate formed with PPARγ and not with PPARα affects the binding affinity. In the future, the activity measurement for PPARα mutants of which residues at the LBP are mutated corresponding to the LBP of PPARγ or vice versa should be analyzed to estimate this point.

The second and more important reason is that pemafibrate can change their conformation due to its flexible phenoxyalkyl group and can make suitable interactions with PPARα (Figure 4). The X-ray structure analysis indicates that electron density map for site A and site B of pemafibrate is clearly observed with or without the binding of SRC1 peptide (Figure 2C,E), whereas the electron density for the site C is disappeared in the absence of the peptide (Figure 2E). By referring to some studies, the characteristic conformational change of PPARα would be induced by the binding of coactivator. Representation is the conformational change at the AF2 interface and Ω-loop. Especially, the physiological importance of the conformational change at Ω-loop is reported for now. In fact, we have indicated earlier that the I272A mutation, which is one of the residues near the Ω-loop completely lost its activity [33]. Notably, the phenoxyalkyl group of pemafibrate is at site B, which includes the Ω-loop. The high flexibility at the phenoxyalkyl group may enable pemafibrate to bind to PPARα strongly with an “induced-fit” mechanism in response to structural changes at site B induced by binding of coactivators. Future works on several mutations of PPARα would help to figure out the mechanism of how pemafibrate can keep this network as a SPPARMα.

## 4. Conclusions

In this study, we succeeded to determine the crystal structure of PPARα-LBD/pemafibrate/SRC1, which indicates that pemafibrate fully occupies the cavity at the LBP of PPARα. The ITC and FMO analysis indicate a strong potency of pemafibrate to PPARα appeared to be brought by the accumulation of weak hydrophobic interactions between the residues at the LBP of PPARα and pemafibrate; this point supports our earlier hypothesis made through combinational approaches of docking simulation and FMO analysis of PPARα-LBD and pemafibrate [33]. The study has also revealed that phenoxyalkyl group of pemafibrate is highly flexible when no coactivator is bound with pemafibrate/PPARα. The binding of the coactivator fixes the conformation of the phenoxyalkyl group as indicated in the crystal structure analysis. This flexible part of the pemafibrate could contribute to the induced-fit feature of the pemafibrate/PPARα complex [33] and the potential coactivator-dependent activation as SPPARMα. These results suggest that the optimum interaction between the drug molecule and the target protein and the flexibility of the molecule is important for the unique features of this novel SPPARMα. It also highlights the usefulness of FMO calculations for predicting the molecular structure of the related functions even before the verification by X-ray structure analysis.

## 5. Materials and Methods

### 5.1. Preparation of PPARs Ligands and Coactivator Peptides

Pemafibrate was provided by Kowa Corporation (Tokyo, Japan). Fenofibric acid was purchased from Tokyo Chemical Industry (Nagoya, Aichi, Japan). These compounds were dissolved into DMSO. The coactivator peptide, SRC1 (687-HKILHRLLQEGS-698), was synthesized by GeneScript (Piscataway, NJ, USA). The peptide was used to perform crystallization and ITC measurement.

### 5.2. Overexpression and Purification of PPARα LBD and PPARγ LBD

A plasmid containing human PPARα LBD (residues 194–468) was cloned into the pET28 vector. These plasmids were transformed to BL21 (DE3) strain. *Escherichia coli* (*E. coli*) cells were cultured at 37 °C in LB medium with 30 μg/mL Kanamycin Sulfate. After that, PPARα LBD expression was induced by adding 0.5 mM of isopropyl β-D-thiogalactopyranoside to the medium and incubated for 48 h at 16 °C. These cells were collected and resuspended into buffer A (20 mM HEPES-NaOH (pH 8.0), 100 mM NaCl, 0.5 mM Tris(2-carboxyethyl)phosphine (TCEP), and 5% (*v/v*) glycerol). After sonication, the insoluble fraction was removed by centrifugation at 10,000× *g* for 30 min. The obtained supernatant was applied to a HisTrap HP column (GE Healthcare) equilibrated with buffer A. The column was washed by buffer A containing 70 mM imidazole. The samples were eluted with buffer A containing 300 mM imidazole. These eluted fractions were concentrated and his-tag was removed by 200 U thrombin, and dialyzed in buffer B (20 mM HEPES-NaOH (pH 8.0), 10 mM NaCl, 0.5 mM TCEP, and 5% (*v/v*) glycerol) using a molecular porous membrane (RERPLIGEN) at 4 °C for two days. Dialyzed samples were concentrated and applied to a MonoQ column (GE Healthcare, Chicago, IL, USA) and eluted with an NaCl gradient (10–500 mM). The eluted fraction was further purified on a gel filtration column (Superdex 200 Increase) (GE Healthcare) equilibrated with bufferA. Fractions containing samples were collected and applied to an SDS-PAGE to check their purity. The samples were utilized in subsequent experiments. Human PPARγ LBD (residues 206–477) were cloned into pET28 vector, and the proteins were expressed and purified by the same procedure described in PPARα LBD.

### 5.3. Crystallization and X-ray Data Collection of the PPARα-LBD/Pemafibrate/SRC1 Peptide

The purified PPARα-LBD samples were concentrated to about 15 mg/mL. The samples which ligate pemafibrate and SRC1 peptide (PPARα-LBD/pemafibrate/SRC1) were obtained by mixing PPARα-LBD, pemafibrate and SRC1 peptide as the following molar ratio: 1:3:5 = PPARα-LBD:pemafibrate:SRC1 peptide. The mixed samples were incubated for 24 h at 4 °C. The crystals of the PPARα-LBD/pemafibrate/SRC1 appeared under the condition of the following reservoir: 1.2 M ammonium sulfate and 0.1 M bis-tris-HCl (pH 6.5) at 22 °C.

The obtained crystals were soaked into cryo-reservoir containing 20% (*v/v*) glycerol and 100 μM pemafibrate in the reservoir solution. The soaked crystals were mounted and flash-cooled under a nitrogen stream (−173 °C). Diffraction data were collected using Dectris Pilatus3 S6M detector at BL5A of Photon Factory (Tsukuba, Japan). The data were collected, scaled, and integrated by HKL2000 and SCALEPACK [39]. The initial phase was determined by the molecular replacement method with MOLREP [40] utilizing the structure for chain A of the complex PPARα/AL26-29 (PDB ID: 5HYK). Model building and structure refinement were performed by COOT [41] and REFMAC [42], respectively. All figures were prepared by PyMOL [43]. The crystallographic table is written in Table 2.

### 5.4. Fragment Molecular Orbital Calculations

The fragment molecular orbital (FMO) calculation for PPARα-LBD/pemafibrate/SRC1 complex was performed by referring to the previous study [44,45,46]. The PPARα-LBD/pemafibrate/SRC1 structure was protonated at pH 7.0 condition by the Protonate3 tool implemented in MOE [47]. Energy minimization was performed by imposing restraint on all atoms in the structure except for the hydrogen atom with molecular mechanics calculations utilizing the Amber10:EHT force-field with solvation energy accounted via the Born model with root mean square deviation (RMSD) gradient setting 0.01 kcal mol^−1^ Å^−2^. By referring to a previous study [48], the structure was divided into one-residue fragments. The fragment assignment and parallelized *ab initio* calculation system based on FMO (PAICS) input generation was done using the free utility PaicsView. The FMO calculation was performed with the PAICS software [49] at the resolution of the identity approximation of the second-order Møller−Plesset perturbation theory (RI-MP2), with the double zeta set of the correlation-consistent polarized valence basis set (cc-pVDZ) level. In this study, we analyzed the interactions between pemafibrate and PPARα-LBD by calculating interfragment interaction energies (IFIEs). The IFIEs were divided into three energy terms: HF, dMP2, and MP2. Here, the counterpoise (CP) correction method [50] was adopted to calculate the IFIEs to avoid the basis set superposition error [51]. All IFIEs were CP-corrected values. The IFIEs between pemafibrate and PPARα-LBD were colored by the RbAnalysisFMO toolkit [52].

### 5.5. Isothermal Titration Calorimetry

The interaction energy between proteins (PPARα LBD, PPARγ LBD) and ligands (pemafibrate, fenofibric acid) were estimated by the ITC experiment. Proteins and ligands were resuspended in bufferA containing 5% (*v/v*) DMSO. The experiment was performed at 25 °C temperature by PEAQ-ITC (Malvern Panalytical Worcestershire, UK). To quantify the interaction energies between proteins and the pemafibrate, the final concentration of the protein and pemafibrate was determined as the following: 500 μM pemafibrate filled in the syringe were titrated into 49–50 μM PPARα LBD, and 50 μM PPARγ LBD in the cell, respectively. In the case of quantification of interaction energies between proteins and the fenofibric acid, the final concentration of the protein and fenofibric acid was set to the following: 500–650 μM fenofibric acid filled in the syringe were titrated into 65–100 μM PPARα LBD, 300–650 μM fenofibric acid filled in the syringe were titrated into 65–100 μM PPARγ LBD in the cell, respectively. The obtained data were analyzed by utilizing the PEAQ-ITC Analysis software (Malvern Panalytical).

## Figures and Tables

**Table 1:** Thermodynamic parameters for the interaction between PPARs-LBD (PPARα-LBD and PPARγ-LBD) and two fibrates (pemafibrate and fenofibric acid) at 25 °C measured by ITC ^a^.

	*n*	*K*_d_	Δ*G*	Δ*H*	*−T*Δ*S*
		μM		kcal/mol	
**Pemafibrate**					
PPARα-LBD	0.61 ± 0.03	0.13 ± 0.04	−9.37 ± 0.02	−12.3 ± 0.6	3.13 ± 0.69
PPARγ-LBD	0.65 ± 0.05	9.58 ± 1.85	−6.83 ± 0.41	−17.1 ± 1.0	10.3 ± 1.2
**Fenofibric Acid**					
PPARα-LBD	0.27 ± 0.04	7.37 ± 2.68	−7.02 ± 0.25	−23.2 ± 2.3	16.1 ± 2.5
PPARγ-LBD	n.d. ^b^	n.d.	n.d.	n.d.	n.d.

^a^ ITC measurement was performed independently three times (*n* = 3). ^b^ n.d. means “not determined”.

**Table 2:** Statistics of X-ray diffraction data collection for PPARα-LBD (194-468) complexed with pemafibrate and coactivator peptide, SRC1.

	PPARα-LBD/Pemafibrate/SRC1
Space group	P3_1_21
Unit cell parameters	
a (Å)	82.74
b (Å)	82.74
c (Å)	177.5
α (degree)	90.0
β (degree)	90.0
γ (degree)	120.0
X-ray source	PF BL5A
Wavelength (Å)	1.00
Resolution (Å)	45.7–3.2 (3.26–3.2)
No. of reflections ^a^	131,418
No. of unique reflections	224,57
Completeness (%)	100 (100)
I/sig(I)	20.8 (1.5)
*R*_merge_^b^	0.080 (0.678)
CC1/2	0.996 (0.800)
*R* ^c^	0.190
*R*_free_^d^	0.253
RMSD of geometry	
Bond length (Å)	0.013
Bond angle (degree)	1.656
Geometry	
Ramachandran outlier (%)	0.4
Ramachandran favored (%)	99.6
PDB code	6L96

^a^ Sigma cutoff was set to none (F > 0σF). ^b^ *R*_merge_ = Σ*_h_*Σ*_i_*|*I_i_*(*h*) − <*I*(*h*)>|/Σ*_h_ I*(*h*), where *I_i_*(*h*) is the *i*^th^ measurement of reflection *h*, and <*I*(*h*)> is the mean value of the symmetry-related reflection intensities. Values in brackets are for the shell of the highest resolution. ^c^ *R* = Σ||*F_o_*| − |*F_c_* ||/Σ|*F_o_*|, where *F_o_* and *F_c_* are the observed and calculated structure factors used in the refinement, respectively. ^d^ *R*_free_ is the *R*-factor calculated using 5% of the reflections chosen at random and omitted from the refinement.

## Abbreviations

PPAR	Peroxisome proliferator-activated receptor
LBD	Ligand binding domain
LBP	Ligand binding pocket
SPPARM	Selective PPAR modulator
FMO	Fragment molecular orbital
ITC	Isothermal titration calorimetry
IFIE	Interfragment interaction energy
